# Declining yet persistent use of traditional contraceptive methods in low- and middle-income countries

**DOI:** 10.1017/S0021932021000341

**Published:** 2021-07-16

**Authors:** Jane T. Bertrand, John Ross, Annie L. Glover

**Affiliations:** 1 Department of Health Policy & Management, School of Public Health & Tropical Medicine, Tulane University, New Orleans, USA; 2 Independent Demographic Consultant, New Paltz, USA; 3 Institute for Global Health & Infectious Diseases, University of North Carolina at Chapel Hill, Chapel Hill, USA

**Keywords:** Traditional contraceptive use, Low- and middle-income countries, Withdrawal, Rhythm, Periodic abstinence

## Abstract

Traditional contraceptive methods are used by 55 million women in developing countries. This study analysed over 80 national surveys to compare traditional with modern method users, by type, region, sociodemographic characteristics, strength of family planning programmes and discontinuation rates. The advance of modern methods has greatly reduced the share held by traditional methods, but the actual prevalence of their use has declined little. Young, sexually active unmarried women use traditional contraception much more than their married counterparts. Discontinuation rates are somewhat lower for traditional methods than for the resupply methods of the pill, injectable and condom; among users of all of these methods, more than a quarter stop use in the first year to switch to alternative methods. Traditional method use is firmly entrenched in many countries, as the initial method tried, a bridge method to modern contraception and even the primary method where other methods are not easily available.

## Introduction

In the last five decades, contraceptive use has increased steadily across low- and middle- income countries (LMIC), with parallel declines in fertility. The contraceptive prevalence rate (CPR, the percentage of married women using a contraceptive method) in LMIC was estimated at less than 10% as of the 1960s. Today, 61% of married women in developing countries use contraception (United Nations, 2019a, 2020). During this same period the average total fertility rate (TFR) declined from 6.04 to 2.56 in the LMIC group of countries (United Nations, 2019a).

The CPR is based on the use of both modern and traditional methods. ‘Modern’ methods include male and female sterilization, intrauterine device (IUD), pill, injectable, implant and condom. (Several other methods, including fertility awareness methods, foam/spermicides, diaphragm and lactational amenorrhoea, are also classified as ‘modern’, but account for an extremely small percentage of method use in most countries.) Traditional methods (TM) include rhythm (also known as periodic abstinence), withdrawal and other folkloric methods. While the modern contraception prevalence rate (percentage using a modern method) (MCPR) far outstrips the use of traditional methods, the latter represent a non-trivial share of total method use.

Withdrawal – a traditional method – played a key role in the first demographic transition, beginning in Europe in the 18^th^ century (Santow, 1995). By contrast, the proliferation of modern methods has been largely responsible for the second transition, dubbed the ‘contraceptive revolution’ (Mathai, 2008; Lesthaeghe, 2010). Whereas modern contraception has eclipsed traditional method use in terms of prevalence and programmatic emphasis, traditional methods continue to play a significant role in fertility regulation (Johnson-Hanks, 2002). A recent analysis of 113 LMIC countries (Bertrand et al., 2020) found 11% of married women to be currently using a traditional method, or about one in ten (population weighted average). It ranked higher as an unweighted average across the countries, at 17% of total use.

Traditional methods have a long history, with references to withdrawal found in ancient religious texts, demonstrating the staying power of these methods. Coitus interruptus is referenced in the Bible with the story of Judah and Onan in Genesis 38:8–9 (van de Walle & Muhsam, 1995). The Talmud referred to withdrawal as ‘threshing inside and winnowing outside’ (Deys & Potts, 1973; Free & Alexander, 1976). The prophet Mohammed reportedly approved of withdrawal (Rogow & Horowitz, 1995), which has a longer history than rhythm. The Vatican did not endorse rhythm until 1951 (Musallam, 1983, as cited by Schneider and Schneider, 1991). In all likelihood, most couples using rhythm or withdrawal are unaware of this history.

From the 1970s to the 1990s, numerous research articles examined the topic of traditional methods (Free & Alexander, 1976; Diller & Hembree, 1977; Logarta-Avila, 1985; Bain, 1989; Maynard-Tucker, 1989; Sheon & Stanton, 1989; Tsui et al., 1991; Mbizvo & Adamchack, 1992; Ringheim, 1993; Bledsoe et al., 1994; Goldberg & Toros, 1994; Rogow & Horowitz, 1995). In the past two decades, the published research on this topic has focused largely on sub-Saharan Africa (SSA): Nigeria (Audu et al., 2006; Garba et al., 2012; Rabiu & Rufa’i, 2018), Ghana (Marston et al., 2017), Burkina Faso (Rossier et al., 2014) and Uganda (Higgins et al., 2014). Exceptions include Iran (Rahnama et al., 2010), Turkey (Ortayli et al., 2005), India (Shahina et al., 2013; Ram et al., 2014) and a multi-country comparison (D’Arcangues, 2001). Recent comprehensive analyses on traditional methods include an article by Rossier and Corker (2017) based on data from 29 countries in sub-Sahara Africa, and a DHS report on traditional use in sixteen countries (Gebreselassie et al., 2017; Rossier & Corker, 2017).

Rossier and Corker (2017) discussed the contribution of abstinence in regulating pre-transitional fertility in sub-Saharan Africa, citing two forms supported by social norms: refraining of sexual intercourse prior to marriage and (more specific to the region) observing relatively long periods of postpartum abstinence. Data on the age at first sexual experience and the age at first marriage suggest wide variations in the extent of premarital abstinence, although it remains a strategy used by some adolescents to avoid pregnancy (Kirby, 2008; Gyan, 2018). Regarding post-partum abstinence, it is one of two historically widespread practices that favoured natural pregnancy prevention and birth spacing in numerous SSA countries, the other being extended breastfeeding (Caldwell & Caldwell, 1977; Page & Lesthaeghe, 1981; Bertrand et al., 1983, 1985; van de Walle & van de Walle, 1989; Garba et al., 2012). To achieve extended post-partum abstinence, the mother would return to her native village, or her mother or mother-in-law would take up residence in the home to prevent sexual contact between the new mother and her husband/partner. In some societies this practice resulted in a separation of 18–24 months, during which time the male partner would be free to have relations with other women, through formal polygamy or informal arrangements. In others, it lasted until the infant could walk. As the practice eroded, the period dwindled to several months. With urbanization, many women preferred to keep their male partners from seeking out other women, so they looked for ways to reduce the period of post-partum abstinence. That in turn led to an interest in methods to avert an early pregnancy, including rhythm and withdrawal.

Methodological studies from Burkina Faso (Rossier et al., 2014), Nigeria (Garba et al., 2012; Rabiu & Rufa’i, 2018) and Ghana (Marston et al., 2017) demonstrated that traditional method use may be underestimated in national surveys such as the DHS, especially if they are used in combination with a modern method (because in these surveys, a women reporting use of both a modern and traditional method is classified as a modern user). Couples may also use multiple traditional methods over the course of a month, depending on their perceived risk of pregnancy and fertility (Tsui et al., 1991).

The persistence of traditional method use – despite the strong emphasis given to modern methods in most national family planning programmes – raises multiple policy and programmatic issues. Key to this dilemma is the lower effectiveness of traditional methods; the 12-month pregnancy rate for withdrawal is estimated to be 20 times higher than implants, IUDs and sterilization (World Health Organization, 2018). What then should be the role of traditional method use? Given the current mantra in the international family planning community of informed choice (facilitating use of the client’s preferred method), some programme managers find themselves in a conundrum. They prioritize more-effective methods, whereas users may prefer traditional methods for various reasons: to avoid negative side-effects of hormonal methods (D’Arcangues, 2001; Ram et al., 2014; Ajayi et al., 2018), including fear of permanent sterility caused by hormonal methods (Sedlander et al., 2018); to experience increased sexual pleasure (Higgins et al., 2014); or for lack of access to modern methods (Ajayi et al., 2018).

The current analysis revisits some of the topics in the references cited above, but updates the findings and expands on our knowledge by including a larger number of countries, on the following topics: i) traditional method use and its share of the method mix in LMIC at the global, regional, and country level; ii) differences in discontinuation rates for resupply methods and traditional methods; iii) changes in prevalence and share of traditional method use over time; and iv) socio-demographic correlates of traditional method use.

This article also addresses new questions not previously analysed for such a large set of countries (or at all): i) estimates of the number of women using traditional methods, as well as the countries with the greatest number of traditional users; ii) relative importance of withdrawal and rhythm by region and by country; iii) traditional use among young women aged 15–24 by marital status; and iv) association between traditional method use and strength of family planning programmes, based on Family Planning Programme Effort Scores.

## Methods

This study used national surveys from 83 low- and middle-income countries in the DHS series, for several reasons (USAID, 2012). The DHS surveys have a relatively common methodology across countries, include a set of personal characteristics of respondents including wealth quintiles and provide data for numerous countries on unmarried sexually active women, again with personal characteristics. Only those surveys with information on the use of eight methods were included: female sterilization, male sterilization (vasectomy), IUD, implant, pill, injectable, condom and traditional methods. The latter fall into three categories: rhythm (also known as periodic abstinence), withdrawal and ‘other traditional methods’ (including folkloric). However, these ‘other’ methods are not systematically included in DHS surveys. A quarter (24.1%) of the 83 latest surveys do not include ‘other’ methods, and in those that do, they represent only half of one per cent prevalence. Therefore, traditional methods have been limited to rhythm and withdrawal.

In several sections of the analysis, the earliest and latest available DHS surveys were used for each country. These surveys were conducted in different years, so the intervals differ, the mean being 17 years between the earliest and most recent (latest) study in these countries. All sections of this analysis, except that on the use of traditional methods among unmarried sexually active women, focused exclusively on women married or in union, aged 15–49 years. The surveys available covered the largest countries in each region (except China), as well as most of the second-largest populations.

Family Planning Programme Effort Scores were used to test the hypothesis that traditional method use is higher in countries with weak national family planning programming, last collected in 2014 and available for 90 countries (Kuang & Brodsky, 2016). The measurement yields 36 individual scores, organized by four categories: policies, services, evaluation and public access to six contraceptive methods plus safe abortion. Collectively, the scores measure the strength of each country’s family planning programme.

The methods employed included cross-tabulations, correlations and graphical presentations. All regional averages were population-weighted (by women aged 15–49). Selected results are presented for six geographic regions; sub-Saharan Africa, with the largest number of countries, is divided into the East/Southern and the West/Central regions since they differ considerably on numerous fertility and family planning indicators.

## Results

### Numbers of traditional method users

In LMIC some 55 million married women currently use traditional methods, by simple multiplication of the numbers of married women by the percentage using in each country. Half of the users (51.1%) reside in just five countries: India with 14.9 million, Iran and Turkey with 3.5 million each and Bangladesh and Pakistan with 3.1 million each. The percentage using traditional methods bears little relation to country size: only 5.7% in India, 8.4% in Bangladesh and 9.2% in Pakistan use traditional methods, compared with 21.7% in Iran and 26.0% in Turkey (latest DHS surveys).

Five additional countries account for large total numbers of traditional method users, despite wide variations among them in method share. In China, a mere 0.6% of contraceptive users are using traditional methods, totalling approximately 1.5 million users due to its large total population size. The Philippines, Vietnam, DR Congo and Indonesia each have between 1.1 and 3.0 million users. Just 2.2% of all contraception users in Indonesia use traditional methods, while the share for the Philippines, Vietnam and DR Congo ranges between 11% and 18%.

### Prevalence of traditional method use by region and country

The use of traditional methods can be measured either as *prevalence* (the percentage of *women* who use the method) or as *share* (the percentage of all contraceptive *users* who rely on traditional methods). This analysis begins with prevalence. As a weighted average across all 83 countries in the analysis, 6.6% of women use a traditional method, while 43.7% use a modern method, totalling 50.3% using some type of method (known as the contraceptive prevalence rate or CPR).

**Figure 1.**
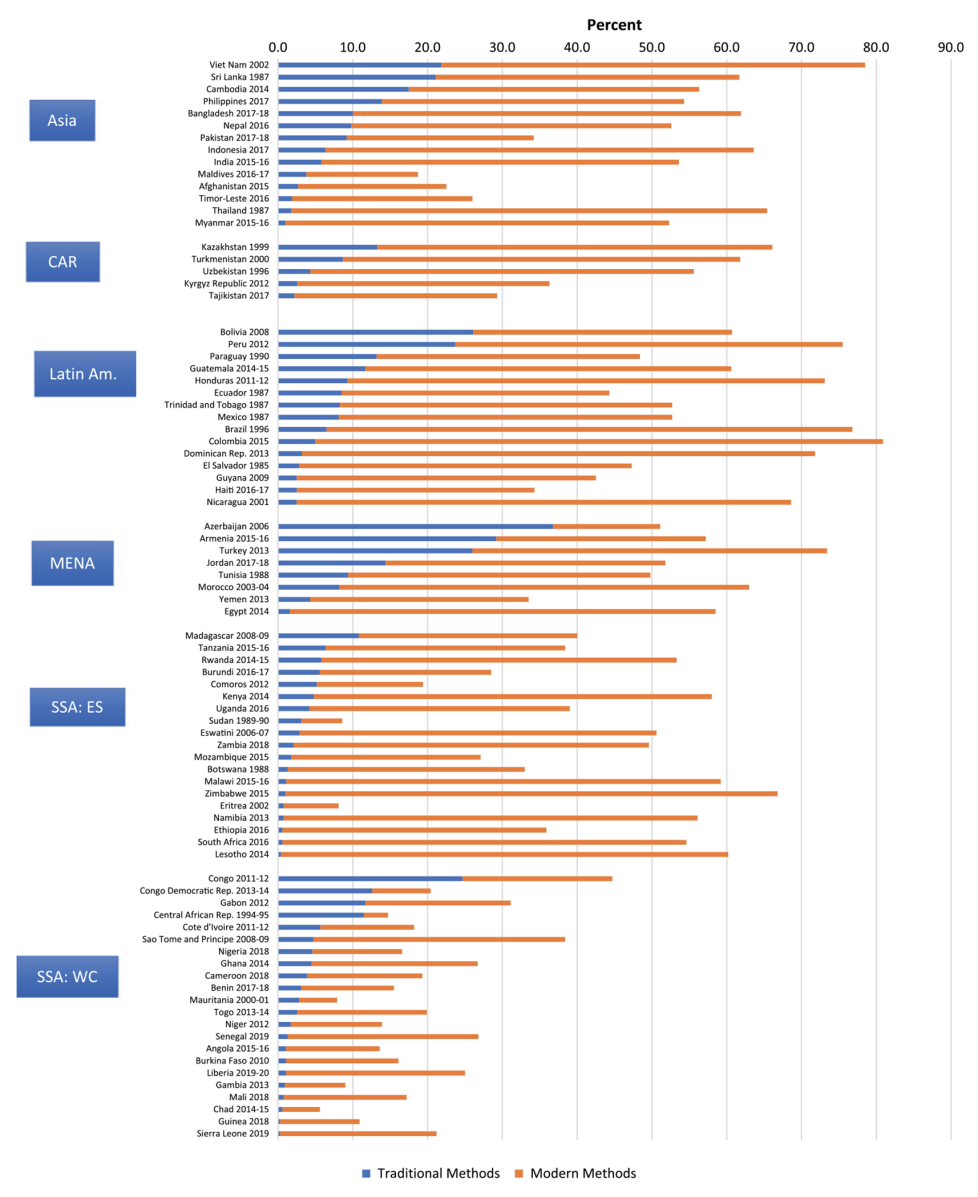
Contraceptive prevalence rates (CPR) divided by traditional vs modern methods. CAR: Central Asia Republics; Latin: Latin America; MENA: Mideast/North Africa; SSA-ES: sub-Saharan Africa-East/Southern; SSA-WC: sub-Saharan Africa-West/Central.

Traditional method use varies markedly by region and by country within each region. Figure 1 presents countries within each region by the level of traditional method use from high to low (left segment in each bar). Of the six regions shown, the prevalence of traditional method use is highest in the Mideast/North Africa (MENA) region; in this region, traditional methods tie with the pill as the most commonly used (Bertrand et al., 2020). Countries heading the list in that region are Azerbaijan (36.5%), Armenia (28.8%) and Turkey (25.8%).

By contrast, the Central Asia Republics (CAR) region reports the lowest levels of traditional method use, due largely to the preference there for the IUD as a modern method (data not shown in Figure 1). In all five countries, less than 15% of married women use a traditional method. Latin America and Asia fall in the middle range for traditional method use, but with large variations by country. In Latin America, Bolivia (25.7%) and Peru (22.6%) show the highest levels of traditional method use. In Asia, it is Vietnam (21.8%) and Sri Lanka (18.3%).

Finally, sub-Saharan Africa presents an interesting contrast between its two major sub-regions. For total use, the average CPR (not shown in Figure 1) is over twice as high in East/South SSA (41.3%) as in West/Central SSA (18.9%); yet traditional method use is lower on average in East/South SSA (2.9%) than in West/Central SSA (4.1%). Countries reporting the highest levels of traditional method use in SSA are Congo-Brazzaville (22.8%) and the Democratic Republic of Congo (DRC) (11.6%).

### Share: the percentage of total use corresponding to traditional methods

In contrast to prevalence (based on all women or all married women), the share is the percentage of all users who rely on a given method. Based on the most recent surveys, weighted by population size, traditional methods make up 14.4% of all use.

**Figure 2.**
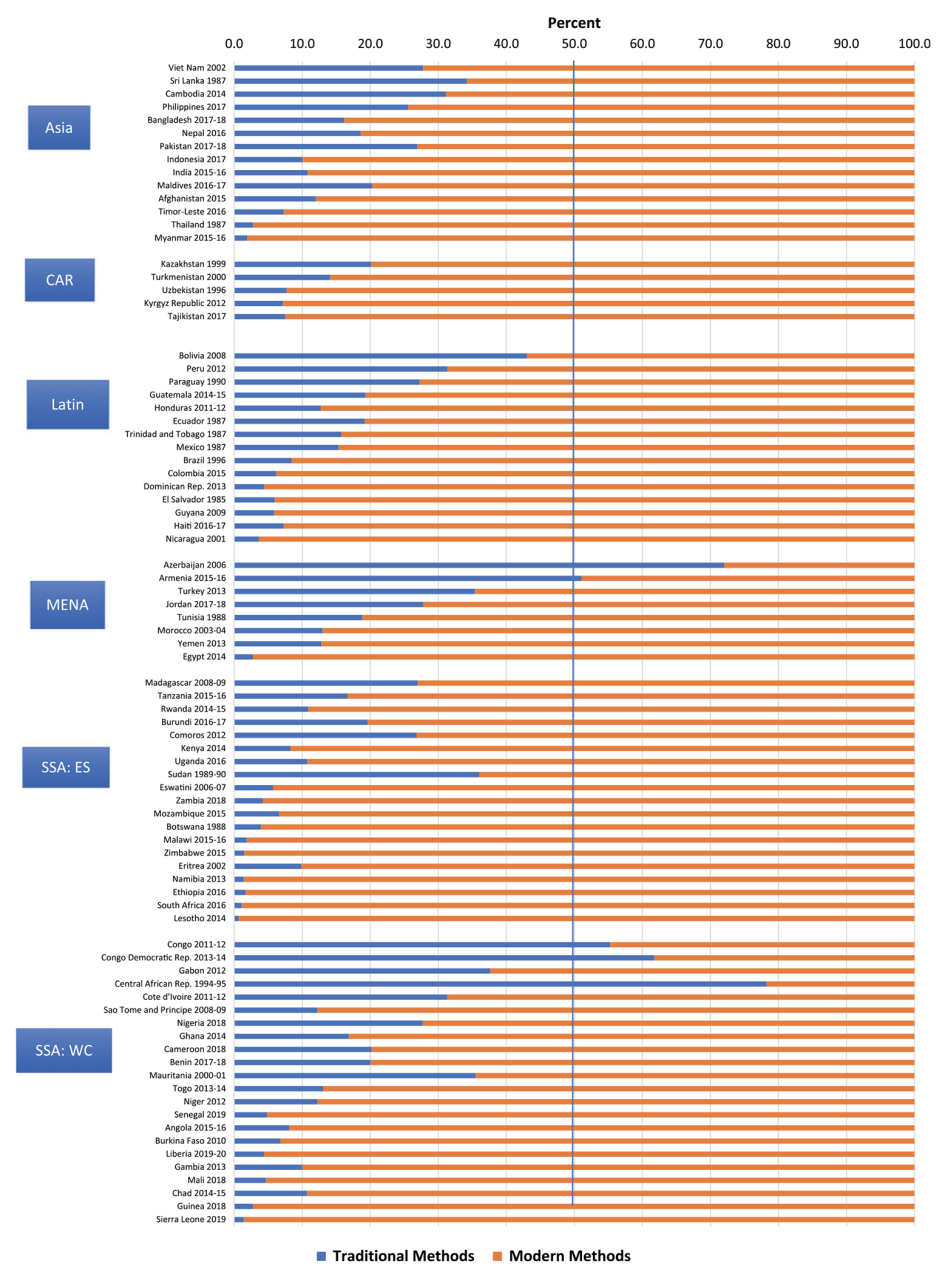
Total contraceptive use divided by traditional vs modern methods.

Figure 2 shows the percentage of all use corresponding to traditional versus modern methods by region and country (in the same order as in Figure 1). The patterns of the bars at the left for traditional methods are more uneven than the ones in Figure 1, since they are affected by the level of modern method use in each country. The different patterns are especially pronounced within sub-Saharan Africa. In the West/Central SSA countries, there is relatively higher dependence on traditional methods, given that the uptake of modern contraception has not proceeded as rapidly as in the East/Southern SSA.

Table 1 provides the average share for traditional method use in each region and the number of countries in each region. Figure 2 shows the countries with a skewed method mix (over 50% of use corresponding to a single method) related to traditional methods: Central African Republic (78.2%), Azerbaijan (72.0%), the DR Congo (61.8%), Congo-Brazzaville (55.3%) and Armenia (51.0). Four non-DHS countries (not shown) also exceed the 50% traditional method share: South Sudan (65.7%), Libya (51.6%), Bahrain (51.3%) and Mauritius (50.7%).

### Decline in traditional method use

There has been a gradual decline in the share that traditional use represents of total use (CPR) in LMIC (Bertrand et al., 2020). Overall, the traditional method share fell from its highest levels of 46.6% (in the 1960s) and 32.1% (in the 1970s), down to 20.8% in the 2000s and 15.3% by the 2010s. In the early years, traditional method use often represented a large share of a ‘pie’ that was very small, and the share declined as the use of modern methods increased. The trend was examined within the 62 countries that had two or more DHS surveys (mean period between surveys 17 years). The declines in traditional method use were common, but by far the greatest in sub-Saharan Africa (37.8% loss), followed by Latin America (15.0%), Asia (12.8%) and MENA (1.2%) (data shown as percentage points). Large declines were due in part to the very high levels of traditional use in early years, especially in many sub-Saharan countries.

**Figure 3.**
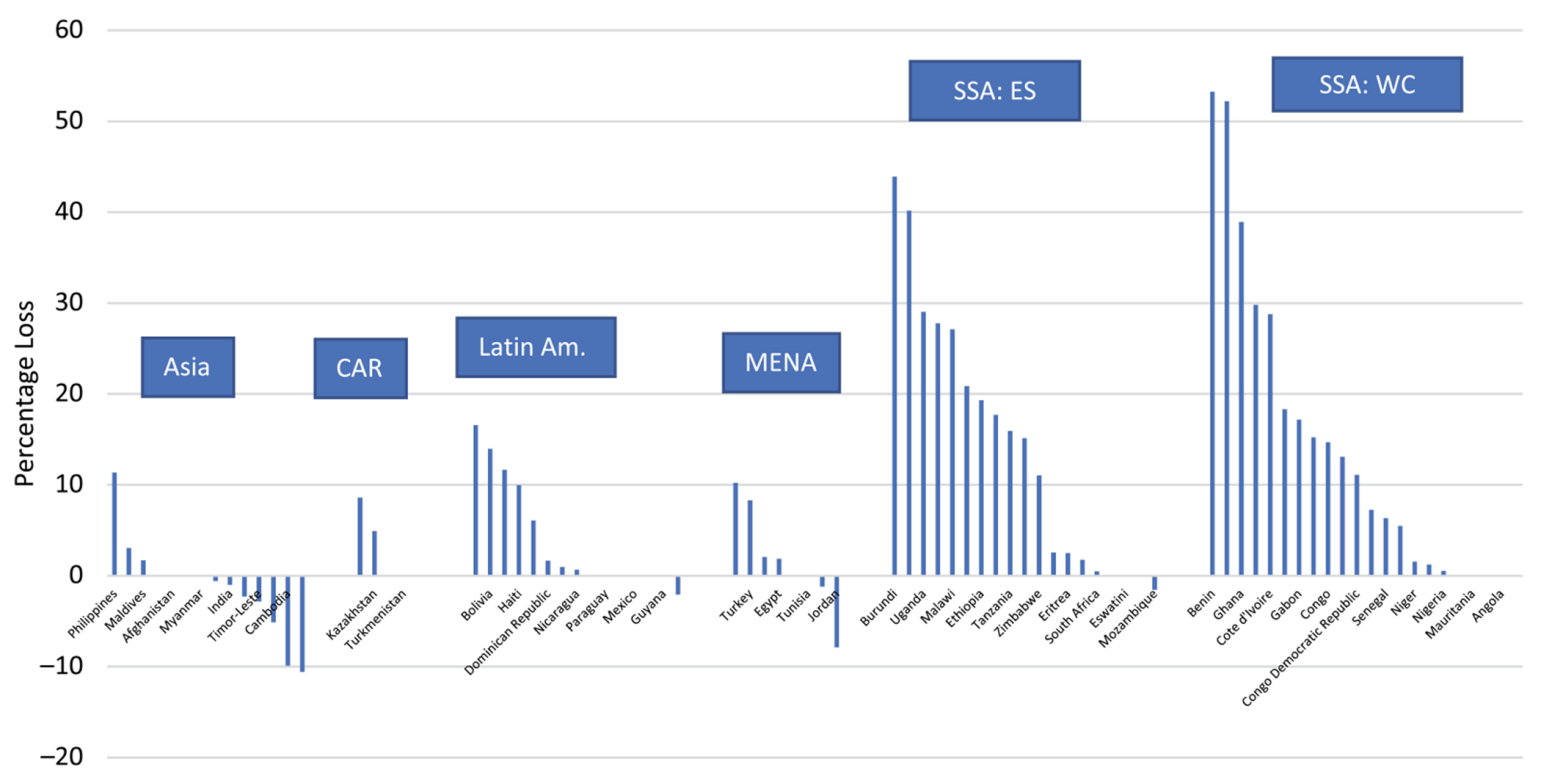
Losses in the traditional method share of all contraceptive use between the earliest and latest surveys, by region and country.

Declines occurred in most of the 62 countries for the traditional method share between the earliest and most recent surveys. As Figure 3 shows, the losses in certain sub-Saharan countries were remarkable. Benin was the extreme case; its share fell from 72.0% to 18.7% – a 53 percentage point drop. Chad, Burundi, Madagascar and Ghana lost about 40 percentage points, indicating that modern methods have increasingly dominated the contraceptive market (all shown as percentage points). By contrast, in about a fifth of the 62 countries examined, traditional methods actually gained share, most notably in Nepal, Cambodia and Jordan.

A decline in traditional methods as a share of method mix results from the net changes between traditional and modern method trends, whether up or down. Here, the use of modern methods has increased much more quickly than that of traditional methods. Prevalence data from the same 62 countries that had two or more DHS surveys were analysed to investigate which of these two trends predominated. As shown in Table 2, on average:

- CPR (modern and traditional) increased from 36.9% to 51.3% (14.4 percentage points)
- MCPR (modern) increased by a greater proportion than CPR from 30.4% to 44.3% (13.9 percentage points)
- Traditional use increased only from 6.2% to 6.7% (0.5 percentage points).

In sum, modern contraceptive use grew rapidly at a time when the percentage of women using traditional methods changed little, creating an ever-increasing gap between the prevalence of modern and traditional methods, causing the traditional method share to plummet.

### Relative Importance of withdrawal versus rhythm by region and by country

To the extent that researchers focus on traditional methods, they tend to combine the two main categories of rhythm and withdrawal (Gebreselassie et al., 2017). Yet for an individual couple, the two methods have very different implications and require different skills and discipline. This section examines the relative importance of withdrawal and rhythm.

Based on the weighted average across all 83 countries, withdrawal and rhythm account for 50% each of traditional method use (Table 3). However, remarkable variation is found among regions and countries in the use of one versus the other of the two methods, as Table 3 shows. The preference for withdrawal is highest in the Middle East/North Africa and in the Central Asia Republics (at about 69%). In Asia, the share is about even (51.5%). Preference for rhythm as a percentage of traditional use occurs in SSA (61.4% in the East/Southern region and 56.8% in the West/Central region) and in Latin America (52.6%).

Curiously, the preference for one over the other may differ across neighbouring provinces of the same country and remain strongly consistent over repeated surveys. In Kinshasa, DR Congo, rhythm was far more popular than withdrawal over six rounds of Performance Monitoring for Action 2020 (PMA2020). By contrast, in Kongo Central, withdrawal was the preferred method over four rounds of PMA2020 (Performance Monitoring and Accountability 2020, 2014–2017).

### Traditional method use by personal characteristics

**Figure 4.**
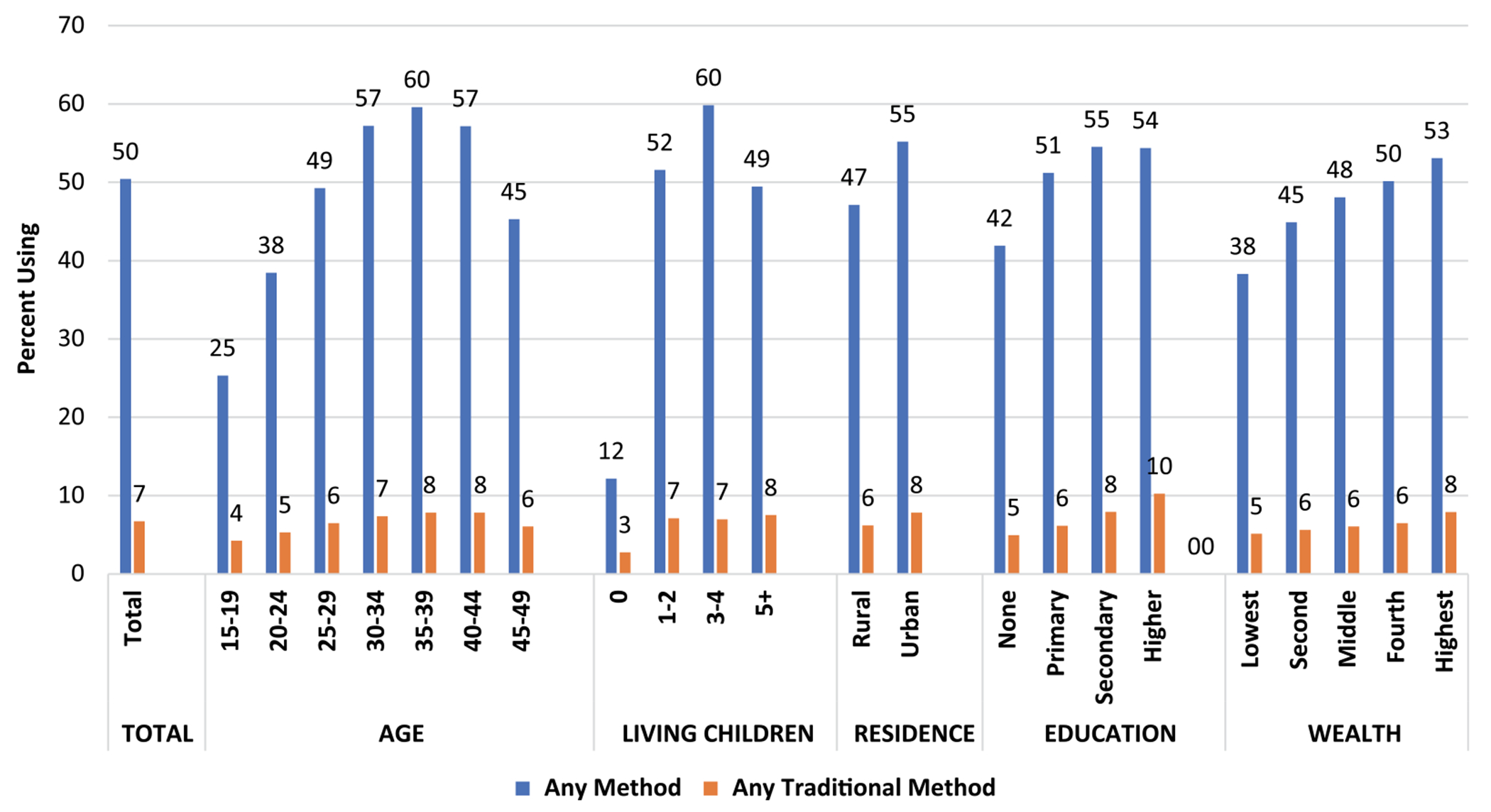
Percentage using any method and any traditional method.

It is of interest to examine differentials of use according to the personal characteristics of women, in terms of both prevalence and share. For prevalence, traditional use is a small proportion of total use, but as Figure 4 shows, the two patterns are similar for the five characteristics; that is, the lines rise and fall together. For age, use increases but then declines, as it does by number of living children. Use is greater in urban than rural areas; it rises sharply with education and also with household wealth quintiles.

**Figure 5.**
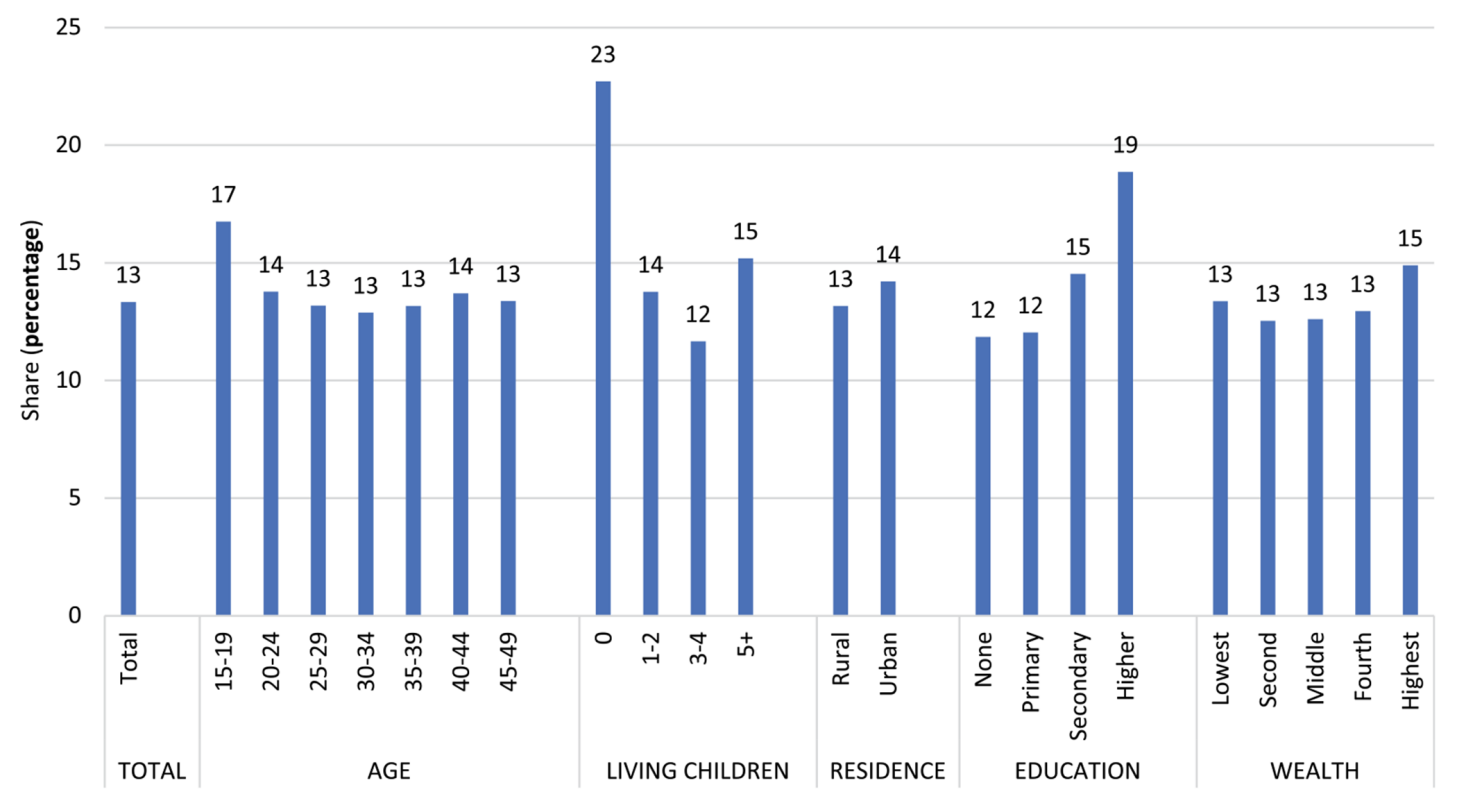
Share of total use held by traditional methods, by socio-demographic characteristics.

However, when method *share* (Figure 5) is examined, interesting differences emerge on three characteristics: age, number of children and education. Older women lean more towards traditional methods than younger women do. Women with no children tilt especially towards traditional methods. The sharpest gradient is by education; the levels of traditional use rise steeply with each additional level of educational attainment. By contrast, there is little association between method share and urban/rural residence or wealth quintile. The finding for wealth quintile is odd, since wealth and education are usually correlated, and more women in the higher wealth quintiles live in urban areas.

### Use of traditional methods among unmarried sexually active women

The above analyses are based on women aged 15–49 years, married or in union. However, the international community increasingly recognizes the needs of young sexually active unmarried women. This section examines their levels of use of traditional methods and changes over time.

Thirty-eight countries provide the data from two or more DHS surveys, allowing trends over time to be assessed, but limiting analysis to three regions: Latin America, East/Southern SSA and West/Central SSA (Table 4). The other regions have too few surveys to obtain reliable time trends, but a brief discussion of a few countries in Asia and in the Central Asia Republics follows. For the 38 countries, the average year of the earliest survey was 1994, the latest 2012, yielding a 16-year span. The results presented in this section pertain to women aged 15–19 and 20–24, beginning with a comparison of traditional method use among three groups: all women, women married or in-union and sexually active unmarried women.

The sexually active unmarried group shows far higher levels of use for all methods, for modern methods and for traditional methods, than do women in the other two groups. Moreover, these patterns hold both for women aged 15–19 and 20–24. By contrast, young married women are less likely to use contraception, since so many are seeking their first child.

Second, the points below illustrated how the use of traditional methods among young, sexually active women has changed over time. The pattern is one of a rising use of modern methods, accompanied by a decreasing reliance on traditional methods. Table 5 captures the data across several dimensions: levels and trends, by age, method and region. The principal findings are:

- *Changing levels of use*. In the earliest surveys the prevalence of traditional methods was much higher than in the latest surveys: 13.7% of the women were using them then, falling by about half to only 7.3% (3^rd^ to last column of Table 5).
- *Marked regional differences*. The overall decline in prevalence occurred largely because of West/Central SSA, where use fell from 22.3% to only 10.5%. West/Central SSA’s initial level of 22.3% was well above that in Latin America (12.7%) and East/Southern SSA (5.4%). Surprisingly, as of the latest surveys, use had increased to be higher among women in Latin America than in East/Southern SSA. With the rapid rise in CPR to high levels in Latin America, rhythm and withdrawal may have been partially eclipsed by modern methods.
- *The age gradient*. Use of traditional methods increased with age. Throughout Table 5, this finding holds true in every comparison except for withdrawal in Latin America. As to changes over time, women aged 20–24 also showed greater declines than those aged 15–19 did. Their proportional shifts to less-traditional method use were much sharper, due most likely to a greater uptake of modern methods.
- *Persistence of rhythm as the preferred traditional method over time*. In both the earliest and latest surveys, more women used rhythm than withdrawal by clear margins, with West/Central SSA being the extreme case. This preference remained, even as the percentage using rhythm by the time of the latest surveys in that region had fallen to only 7.4%.

### Relation of traditional methods to programme effort

Family planning programmes have given considerably more attention to modern contraceptive methods than to traditional methods over recent decades, and international agencies have encouraged this emphasis, as in the Sustainable Development Goals (SDG) of the United Nations to increase the numbers of modern method users by 2030 (United Nations, 2015). The efforts of family planning programmes have most likely contributed to the greater growth in modern than in traditional methods, thereby raising the share of modern method use.

To test this hypothesis, the association was tested between the Family Planning Programme Effort Scores that measure the strength of programmes across four dimensions (policies, services, evaluation and access) and the share of total use corresponding to traditional methods. Table 6 shows the correlation matrix between the Family Planning Programme Effort Scores (total score and the four sub-scores) matched to the share of use for any traditional method, withdrawal or rhythm.

Consistent with expectation, there is a negative correlation between the total effort score and traditional method use (−0.35). All relationships are in the expected direction, showing smaller shares of traditional use where programme effort is stronger. Moreover, the correlation is stronger for the categories of ‘services’ and ‘access’ than for ‘policies’ and ‘evaluation’. The correlations are generally similar for total traditional use and for withdrawal, though somewhat smaller for rhythm.

As a further check, the correlations in Table 6 were re-run to explore the relation of the effort scores to traditional prevalence rather than share. In every cell of Table 6 the direction (sign) of the correlation is identical, suggesting that under stronger programmes, TM prevalence is less. However, the correlations are weaker at only about two-thirds of the Table 6 values, probably because non-programme determinants play larger roles for the levels of TM use. Correlation does not demonstrate causation, but the findings are consistent with the programmes contributing to the shift towards modern methods and away from traditional methods.

### Comparisons of discontinuation rates for resupply methods and traditional methods

Traditional methods are often criticized for having high discontinuation and failure rates (Polis et al., 2016), so it is of interest to compare the rates to those for the resupply methods of the pill, injectable and condom. Table 7 presents the first-year discontinuation rates for five methods, based on the 57 latest DHS with relevant data (population weighted). The findings indicate a mixed picture for discontinuation (the estimated proportion of all those starting on a method who discontinue it within one year, regardless of any experience with other methods during the previous five years).

Withdrawal and rhythm show lower discontinuation rates than any of the resupply methods, for the total of ‘all reasons’ for discontinuation, at 36.0% and 30.4%. However, withdrawal and rhythm show much higher discontinuations for method failure, at 6.4% and 6.2%, than the resupply methods do.

Traditional methods, by their inherent character, show nearly no discontinuation because of side-effects and health reasons, whereas these run as high at 12.5% and 16.5% for the pill and injectable. Condoms do nearly as well as the traditional methods in this respect. Switching is shown in Table 7 as a separate category, outside of the ‘All reasons’ row, and not part of the total. That is, women who stop use may or may not switch to another method. The percentage switching is lowest for rhythm (7.0%) in comparison to the other four methods (that round to 10–11%).

In sum, traditional methods compare favourably to the three resupply methods in this analysis in terms of discontinuation, except for their higher levels of method failure.

## Discussion

The above analysis serves to update our understanding of the trends in traditional method use, based on a larger set of countries than previously analysed, and it provides new insights into the dynamics of traditional method use. The essential findings are:

- *The persistence of the prevalence of traditional method use, despite rapid increases in modern use*. While the widespread uptake of modern methods has greatly reduced the relative share held by traditional methods, their actual prevalence of use has changed little (currently at 6.7% or one in fifteen married women, based on weighted data).
- *The near equal proportion of tradition method users who rely on rhythm versus withdrawal*. Currently, rhythm and withdrawal account for 50% each of total traditional method use level, consistent with the findings in a 2013 review that the two were nearly equal in extent of use (Darroch & Singh, 2013). However, there are large regional and country disparities in the use of one over the other. Since the 1970s, researchers have explored reasons for these differences. Rhythm is often more common than withdrawal among couples with higher education levels due to the complexities of tracking fecundity during the monthly cycles (Caldwell et al., 1987; Goldberg & Toros, 1994; Lesthaeghe, 2010). There is significant variability in rhythm users’ understanding of how to effectively practise rhythm, due to lack of knowledge about reproductive physiology (Maynard-Tucker, 1989), lack of knowledge of individual variation in menstrual cycles (Mastroianni, 1974) and inability to identify fertile days (Logarta-Avila, 1985; Sheon & Stanton, 1989; Becker & Costenbader, 2001; Shahina et al., 2013). Withdrawal is free from these problems and has a long history in certain regions, but it requires a special discipline on the part of the male partner.
- *Marked variation in the use of traditional method use based on five personal characteristics: age, family size, education, residence and wealth*. The patterns echo many of those in the recent analysis of sixteen countries with a long series of DHS surveys (Gebreselassie et al., 2017). The variations are sufficiently marked to merit close attention in each country for programme planning, especially as they parallel those for the modern methods.
- *Lower discontinuation rates for traditional methods than for resupply methods (pill, injectable and condom), but higher failure rates*. The perceived harmful side-effects associated with hormonal methods are not an issue for traditional users. However, they are more likely than users of resupply methods to report method failure. Switching to alternative methods occurs by about 10% of those who discontinue within the first year of use. This ‘churning’ among methods goes on constantly, as women and couples seek the most suitable method for their changing circumstances and needs.
- *Higher use of traditional methods among single, sexually active 15- to 24-year-old women than their married counterparts*. The same is true of modern methods, indicating a strong felt need among this subgroup of women to avoid pregnancy with whatever methods they can find or opt to use.
- *The association between traditional method use and weak family planning effort scores*. The study findings support the hypothesis that traditional use often results from lack of access to modern methods. Where family planning programmes are stronger (as measured by the Family Planning Programme Effort Scores), the shares of all use corresponding to traditional methods are smaller. In practice, use of the various modern and traditional types coexist, with switching among methods over time. Therefore, country programmes should seek more positive ways to build on some clients’ desire to use traditional methods in the context of the primary attention to modern methods

In sum, traditional methods are, and will continue to be, used by millions of women. They are rooted deeply in many cultures, and they will play an important role in pregnancy prevention in many countries. Many women prefer traditional methods; others use them as a transition to modern methods; and for some, a traditional method is the only option where access to modern methods is limited.

Policymakers, donors and programme managers have various perspectives on the role that traditional methods can and should play in a national family planning programme. Many family planning specialists marginalize traditional methods as being of lower effectiveness and desirability than modern methods (Jaramillo-Gomez & Londono, 1968; Rossier & Corker, 2017); they characterize them as regressive and counter to progression in the ‘fertility transition model’ (Johnson-Hanks, 2002).

A second group sees value in traditional methods as a bridge towards modern contraception, especially after women experience an unplanned pregnancy while using one. This connection deserves further research. A third group considers traditional methods as important in their own right, for motivated women who dislike modern methods or lack ready access to them. Some have called for traditional methods to be given equal attention in contraceptive programming, not just as a default in the absence of modern ones. That is, as an option with high acceptability for many, hence deserving equal consideration for efficacy in contraception programming (Santow, 1993; Jones et al., 2009; Rossier & Corker, 2017).

It is in the definition of unmet need that the aversion to traditional methods is most evident in the international family planning community. All users of traditional methods are included in the unmet need group, which is defined as the need for *modern* methods. This is the specification in such seminal documents as the Family Planning 2020 (FP2020) progress reports (FP2020, 2020), United Nations Sustainable Development Goals (United Nations, 2019b), and the Guttmacher Institute’s Adding it Up (Darroch et al., 2017). The ‘demand satisfied’ metrics also de-emphasize traditional methods (Gebreselassie et al., 2017).

A related question concerns the role of traditional methods in assessing universal access to reproductive health care. Should traditional methods ‘count’ as covering the needs of women to control their fertility, if they are less effective than modern methods? The answer is not straightforward: in a country with low traditional method use, the question has little relevance, since almost all use is of modern methods, and the greater the extent of traditional method use, the more relevant the question. Failure rates are markedly higher among traditional methods than among resupply methods or long-acting and permanent methods (Polis et al., 2016). The protection they confer is lower than by modern contraceptives, and the individual user of a traditional method runs a greater risk of unintended pregnancy. Yet many women and couples may choose a traditional method over no method. To count traditional methods improves indicators of universal access, but involves more unplanned pregnancies, some of which will end in abortion.

The issue of how to classify traditional method use underscores the tension between a purely woman-centred policy versus attention to demographic trends, as with the demographic dividend, a societal benefit that emerges from declining fertility rates. The dividend results from a shift in the age distribution away from the young and towards the working ages, and it depends upon a declining trend in new entries at the bottom of the age pyramid. In recent years, the international family planning community has given far greater emphasis to client-centred programming than to achieving specific demographic objectives, yet numerous countries tend to view the demographic dividend as an essential means towards economic advancement.

Finally, with the partial marginalization of traditional methods by family planning programmers and researchers, there is a dearth of literature on the motivations for and dynamics of their use. A few studies have documented reasons for using traditional methods, but questions remain: How do women and couples assess the differing advantages of rhythm and withdrawal? To what extent is a traditional method simply a default option when access to modern contraception is limited? In using rhythm, do many women abstain from sex during the fertile periods or revert to condom use? How much do couples communicate openly about the woman’s fertile period? What cultural and religious forces are in play, given the marked regional differences in all of the findings reported here? Despite all the data presented in this article, the need continues for in-depth research to improve our understanding of the dynamics of traditional method use.

## Figures and Tables

**Table 1.** Traditional method use as a share of all use, by region

	Traditional method share (%)	No. of countries
Asia	13.1	14
Central Asia Republics	8.5	5
Latin America	12.3	15
Middle East/North Africa	18.6	8
SSA-East/Southern	8.8	19
SSA-West/Central	26.6	22
Total	14.4	83

**Table 2.** Percentage using traditional and modern methods of contraception in the earliest and latest surveys (62 countries)

	CPR^a^	Modern (%)	Traditional (%)
Earliest surveys	36.9	30.4	6.2
Latest surveys	51.3	44.3	6.7
Change between surveys	14.4	13.9	0.5
No. of countries increasing	49	58	19
% of the 62 countries increasing	79.0	93.5	30.6

a The CPR is slightly greater than the sum of the MCPR and TM due to its inclusion of folk and other methods.

**Table 3.** Relative shares held by withdrawal and rhythm methods of total traditional method use (83 countries)

	Withdrawal (%)	Rhythm (%)	Total (%)
Asia	51.5	48.5	100
Central Asia Republics	69.0	31.0	100
Latin America	47.4	52.6	100
Middle East/North Africa	68.7	31.3	100
SSA-East/Southern	38.6	61.4	100
SSA-West/Central	43.2	56.8	100
Total	50.0	50.0	100

**Table 4.** Percentage using contraception by age, method type and region, 38 countries

	All methods		Modern methods		Traditional methods	
			
	15–19	20–24	15–19	20–24	15–19	20–24
All 3 regions						

All women	11.8	32.4	10.4	28.9	1.4	3.5

Married women	29.1	39.7	26.0	35.9	3.1	3.9

Sexually active	48.8	59.1	43.0	51.0	5.8	8.2

Latin America						

All women	16.8	46.1	15.1	42.0	1.8	4.1

Married women	55.0	66.8	48.4	60.9	6.7	6.0

Sexually active	68.8	81.2	61.0	71.9	7.8	9.3

SSA-East/Southern						

All women	12.3	36.5	11.6	34.3	0.8	2.1

Married women	30.0	43.0	28.6	40.8	1.3	2.2

Sexually active	48.0	59.7	45.5	53.5	2.6	6.2

SSA-West/Central						

All women	7.4	17.6	5.6	13.3	1.8	4.2

Married women	8.0	15.3	5.9	11.4	2.1	3.9

Sexually active	34.0	41.3	26.6	32.0	7.4	9.3

**Table 5.** Trends in percentage using traditional methods among unmarried sexually active women: changes between earliest and latest surveys, 38 countries

		Withdrawal			Rhythm			Any traditional method		
				
		Total^a^	15–19	20–24	Total^a^	15–19	20–24	Total^a^	15–19	20–24
All 3 regions	Earliest	2.4	2.3	2.8	11.3	10.0	13.7	13.7	12.3	16.5

	Latest	2.5	2.3	3.1	4.8	3.5	5.1	7.3	5.8	8.2

	Change	0.2	0.0	0.3	−6.5	−6.5	−8.6	−6.4	−6.5	−8.3

Latin America	Earliest	4.1	3.9	3.1	8.6	5.8	7.4	12.7	9.7	10.5

	Latest	3.7	5.0	4.2	4.0	2.8	5.0	7.7	7.8	9.3

	Change	−0.4	1.1	1.2	−4.6	−3.0	−2.4	−5.0	−1.9	−1.2

SSA-East/Southern	Earliest	0.4	0.5	0.8	5.0	3.4	8.2	5.4	3.8	9.0

	Latest	1.0	0.5	2.5	2.6	2.1	3.7	3.6	2.6	6.2

	Change	0.6	0.0	1.7	−2.4	−1.3	−4.5	−1.8	−1.2	−2.8

SSA-West/Central	Earliest	2.9	2.9	4.5	19.4	19.6	24.0	22.3	22.5	28.5

	Latest	3.1	2.2	3.1	7.4	5.2	6.2	10.5	7.4	9.3

	Change	0.2	−0.6	−1.4	−12.0	−14.5	−17.8	−11.8	−15.1	−19.2

a ‘Total’ in the DHS tabulations pertains to all three age groups (15–19, 20–24 and 25–29 years), so this column only approximates the total for the two age groups used here.

**Table 6.** Correlations between Family Planning Programme Effort Scores and the share of contraceptive use due to traditional methods, 68 countries

	Total score	Policies	Services	Evaluation	Access
Any traditional method	−0.35	−0.28	−0.37	−0.29	−0.30
Withdrawal	−0.34	−0.27	−0.38	−0.32	−0.22
Rhythm	−0.21	−0.17	−0.20	−0.11	−0.24

Correlations are ‘*r*’ values

**Table 7.** First year discontinuation rates by reason and method (57 countries)

	Pill	Injectable	Condom	Withdrawal	Rhythm
Method failure	3.7	1.9	3.4	6.4	6.2
Side-effects, health	12.5	16.5	2.8	1.0	0.7
Desire to become pregnant	9.1	6.7	10.0	8.5	8.4
Other fertility-related reasons	5.4	3.3	6.2	5.1	3.6
Wants a more-effective method	3.0	3.4	4.3	5.5	4.1
Other method-related reasons	3.6	4.6	5.4	3.1	2.3
Other reasons	5.1	6.1	8.0	6.4	5.0
All reasons for above	42.4	42.4	40.3	36.0	30.4
Switching to another method	9.8	11.1	10.7	9.8	7.0
